# Collaborating with our community to increase code sharing

**DOI:** 10.1371/journal.pcbi.1008867

**Published:** 2021-03-30

**Authors:** Lauren Cadwallader, Jason A. Papin, Feilim Mac Gabhann, Rebecca Kirk

**Affiliations:** 1 PLOS, Cambridge, United Kingdom; 2 Department of Biomedical Engineering, University of Virginia, Charlottesville, Virginia, United States of America; 3 Department of Biomedical Engineering and Institute for Computational Medicine, Johns Hopkins University, Baltimore, Maryland, United States of America

PLOS Computational Biology was launched in 2005 as a journal driven by the computational biology research community and with the principle that “open access ensures not only that everything we publish is immediately freely available to anyone, anywhere in the world, but also that the contents of this journal can be redistributed and reused in ways that increase their value.”[1] Delivering this important open vision has relied on the enthusiasm and integrity of the broader computational biology community as editors, as reviewers, and as dedicated authors submitting excellent work to the journal. This vision of openness, redistribution, and reuse is so essential because most scientific progress builds on prior efforts. This progress is possible because as scientists we share not just our research results, but also our methods and protocols and key research tools. Open sharing allows others to check and reproduce our observations, and to build on our work, giving it even more impact over time. This value of sharing is not just true in experimental work, where a key antibody or plasmid must be shared to enable scrutiny and further research; it is also true in computational biology, where computational code is a key reagent. Given how essential newly developed code can be to computational biology research we have been collaborating with the Editorial Board of PLOS Computational Biology and consulting with computational biology researchers to develop a new more-rigorous code policy that is intended to increase code sharing on publication of articles.

Code sharing is not new to many of our authors, and in 2019 over 40% of research articles published in the journal reported sharing some code. [2] Assuming this percentage to be a baseline of code sharing behaviour, and after consulting with individual researchers about their code sharing practices, we surveyed our authors and others working in the computational biology field. The objective of this survey was to determine what proportion of articles have code associated with them, and what proportion of this code has not been openly shared in the past. We also aimed to better understand challenges researchers have to overcome to share their code, and how a stronger code sharing policy might affect their opinion of the journal. [3] The results indicated that around 70% of articles have code associated with them based on the cohort who completed the survey. However, for about a third of these articles the authors have not shared the code in the past. The reasons given for this discrepancy range from practical issues, such as not having enough time, to legal and ethical reasons, which is supported by previous research into code sharing. [4] Analysis of the survey data suggested around 5% of articles could not share code for legal and ethical reasons, and therefore more articles published in PLOS Computational Biology could share code than have to date.

While there are legitimate restrictions on the sharing of some code, we know that availability of code assists with the reproducibility of research, and journal policies are effective solutions in enabling access to research outputs. [5] Taking these elements together we have developed the new code policy for the journal, which will be applicable for articles submitted from 30th March 2021. The policy will require authors to publicly share any code that they created and that directly relates to the results described in the paper, unless there are legal or ethical restrictions on sharing the code. Such exemptions can be requested during submission. Although we support the process by making recommendations and encouraging best practice, the final version of the policy importantly still empowers our authors with the choice of how to share their code, what license to use, and when during the publication process to share—as long as the code is shared upon publication of the paper. And to support transparency and potential reuse, authors will be required to include a statement on code sharing within the data availability statement on each manuscript.

The development or adaptation of computational code is central to research in computational biology and for this reason it is essential that it be made available to other researchers. As academic journals, we have a responsibility to support open research in this area by developing robust and rigorous policies and providing our authors with clear guidelines and support to uphold them. The creation of this policy has been a partnership between the PLOS Computational Biology Editorial Board, members of the computational biology community who gave their time and feedback in interviews, those who completed the survey, and PLOS. The policy is designed to increase code sharing in our journal, and we intend to report on progress towards this goal. By implementing this policy we hope to facilitate more openness and reproducibility in research, and to accelerate scientific discovery through more (and more efficient) code reuse. We will continue to work in partnership with our authors to respond to the needs and goals of their research, and we look forward to the many applications and use cases of the code shared as part of our published papers.

## References

[1] Bourne PE, Brenner SE, Eisen MB. PLoS Computational Biology: a new community journal. PLoS Comput Biol. 2005 6;1(1):e4. 10.1371/journal.pcbi.0010004 16103905 PMC1183510

[2] Boudreau M, Poline J-B, Bellec P, Stikov N. On the open-source landscape of PLOS Computational Biology. PLoS Comput Biol. 2021 2 11;17(2):e1008725. 10.1371/journal.pcbi.1008725 33571204 PMC7877734

[3] Harney J, Hrynaszkiewicz I, Cadwallader L. Code Sharing Survey 2020—PLOS Computational Biology. Figshare 10.6084/m9.figshare.13366025

[4] AlNoamany Y, Borghi JA. Towards computational reproducibility: researcher perspectives on the use and sharing of software. PeerJ Computer Science 2018 4:e163 10.7717/peerj-cs.163 PMC7924683 33816816

[5] Stodden V, Seiler J, Ma Z. An empirical analysis of journal policy effectiveness for computational reproducibility. Proc Natl Acad Sci USA. 2018 3 13;115(11):2584–9. 10.1073/pnas.1708290115 29531050 PMC5856507

